# Metabolic dysfunction and GZMK^+^ CD8^+^ T-cell remodeling in hepatocellular carcinoma: an evidence-stratified, hypothesis-generating review

**DOI:** 10.3389/fimmu.2026.1963310

**Published:** 2026-09-17

**Authors:** Qianqian Zhong, Wanruo Lyu, Jieying Li, Yiming Song, Yunxin Huang, Chunbin Wang, Juling Ji

**Affiliations:** 1 Department of Pathology, Medical School of Nantong University, Nantong, Jiangsu, China; 2 Department of Oncology, The Sixth Affiliated Hospital of Nantong University, Yanchen, Jiangsu, China

**Keywords:** CD8+ T cells, GZMK, hepatocellular carcinoma, immunometabolism, tumor microenvironment

## Abstract

Type 2 diabetes mellitus and metabolic dysfunction associated steatotic liver disease contribute to the growing burden of hepatocellular carcinoma, yet their effects on hepatic antitumor immunity remain incompletely defined. Recent single-cell and spatial profiling studies indicate that hepatocellular carcinoma contains diverse CD8^+^ T cell states, including cytotoxic, exhausted, tissue-resident, progenitor-like, and transitional phenotypes. This review synthesizes evidence linking hyperglycemia, insulin resistance, lipotoxic injury, chronic inflammation, fibrosis, altered antigen presentation, stromal remodeling, chemokine signaling, and nutrient availability to CD8^+^ T-cell remodeling in HCC arising in the context of metabolic dysfunction. Particular attention is given to GZMK^+^ CD8^+^ T cells, which may reflect non-terminal differentiation, inflammatory adaptation, or transitional immune states rather than conventional cytotoxic effector function. Although GZMK-high CD8^+^ T cell populations have been reported in hepatocellular carcinoma and therapy-associated immune contexts, direct evidence that type 2 diabetes induces these cells in the liver remains limited. This review specifically examines GZMK^+^ CD8^+^ T-cell states within the immunometabolic context of HCC, distinguishes direct hepatic and tumor evidence from extrapolated findings in metabolic liver disease and other inflammatory conditions, and proposes a testable framework for phenotypic, spatial, and functional validation. Integrated single-cell, spatial, protein level, T cell receptor clonality, functional, and clinical studies are needed to define their biological significance and translational value.

## 1 Introduction

Primary liver cancer, predominantly hepatocellular carcinoma (HCC), remains a major cause of cancer-related mortality worldwide (1). As the etiological profile of HCC shifts, metabolic disorders—including type 2 diabetes mellitus (T2DM), metabolic dysfunction-associated steatotic liver disease (MASLD), and metabolic dysfunction-associated steatohepatitis (MASH)—are contributing increasingly to disease development (2–4). T2DM is associated with an elevated risk of HCC, while MASH-associated HCC may differ from virus-related disease in its immune contexture and therapeutic response (3–6). How metabolic stress reshapes the liver immune microenvironment during HCC development therefore remains an important unresolved question.

CD8^+^ T cells are central to antitumor immunity, but their behavior in metabolic liver disease and HCC extends beyond a simple progression from activation to terminal exhaustion (6, 7). Single-cell studies have identified multiple interconnected effector, resident, transitional, and exhaustion-related states (7, 8). Within the metabolically injured liver, lipid excess, hyperglycemia, oxidative stress, and persistent inflammation may alter CD8^+^ T-cell differentiation, localization, and function (5, 6). However, the CD8^+^ T-cell states linking metabolic injury to HCC-associated immune remodeling remain poorly defined.

Granzyme K (GZMK)-expressing CD8^+^ T cells have emerged as a potentially relevant intermediate population. These cells often exhibit effector-memory-like and non-terminally exhausted features and differ from conventional GZMB^+^ cytotoxic CD8^+^ T cells in both phenotype and function (9, 10). They have been associated with inflammatory signaling, tissue interactions, and microenvironmental remodeling rather than exclusively with direct target-cell killing (9–11). Their presence in chronically inflamed tissues raises the possibility that they represent a metabolically responsive CD8^+^ T-cell state relevant to liver injury and HCC. However, direct evidence linking T2DM specifically to hepatic GZMK^+^ CD8^+^ T-cell remodeling remains limited, and much of the mechanistic evidence is derived from MASLD/MASH and other metabolically stressed or inflammatory liver settings.

Metabolic dysfunction encompasses overlapping but non-equivalent conditions, including T2DM, obesity, MASLD, and MASH (3, 4, 12–14). Accordingly, this review considers T2DM as one clinically relevant metabolic context of HCC while distinguishing HCC occurring in patients with T2DM from MASLD/MASH-associated HCC and separating direct T2DM-specific evidence from mechanistic extrapolation across related metabolic liver diseases. Within this framework, the proposed relationship between T2DM-associated metabolic stress and GZMK^+^ CD8^+^ T-cell remodeling is treated as a hypothesis-generating model rather than an established T2DM-specific pathway.

Previous reviews have addressed the broader biology and context-dependent functions of GZMK^+^ CD8^+^ T cells across inflammatory and tumor settings, as well as the immune and metabolic microenvironment of steatotic or MASH-associated HCC (8, 15–17). The present Review differs by focusing specifically on the unresolved intersection among metabolic dysfunction—particularly T2DM, GZMK-associated CD8^+^ T-cell states, and HCC. Rather than presenting this relationship as an established mechanistic axis, we distinguish direct disease-specific evidence from cross-context extrapolation, stratify individual propositions according to evidential strength, and identify the phenotypic, spatial, functional, and interventional studies required to test the major unsupported transitions. The added value of this Review therefore lies in providing an evidence-gap and hypothesis-testing framework for this specific intersection, rather than another general overview of GZMK^+^ CD8^+^ T-cell biology or HCC immunometabolism.

## 2 Literature search and evidence classification

### 2.1 Literature search and study selection

A structured literature search was conducted in PubMed/MEDLINE, Web of Science, and Scopus from database inception to August 2026. Search terms were organized around four major concept domains: (1) hepatocellular carcinoma (“hepatocellular carcinoma” OR “HCC” OR “liver cancer”); (2) metabolic dysfunction (“type 2 diabetes” OR “T2DM” OR “diabetes” OR “MASLD” OR “MASH” OR “NAFLD” OR “NASH” OR “metabolic dysfunction” OR “metabolic stress”); (3) CD8^+^ T-cell states (“CD8 T cell” OR “T-cell exhaustion” OR “effector memory” OR “tissue resident” OR “Tpex”); and (4) GZMK-associated biology (“GZMK” OR “granzyme K”). Additional targeted searches were performed for mechanistic topics relevant to the proposed framework, including CXCR4–CXCL12 signaling, extracellular GZMK, complement activation, metabolic regulation of T-cell differentiation, stromal remodeling, and immune checkpoint therapy. Reference lists of key primary studies and relevant reviews were also screened to identify additional eligible publications.

Priority was given to original human studies, single-cell and spatial profiling studies, clinical cohorts, and mechanistic experimental studies directly addressing HCC, metabolic liver disease, CD8^+^ T-cell states, or GZMK biology. Studies from other tumor or inflammatory disease contexts were included only when they provided mechanistic information relevant to a specific biological relationship for which direct evidence in metabolically altered liver disease or HCC was unavailable. Reviews were used primarily to contextualize the field and identify relevant primary literature rather than as the principal basis for mechanistic claims. Studies were excluded when they did not provide information relevant to the predefined metabolic, immunological, or GZMK-associated relationships considered in this Review.

### 2.2 Evidence-directness classification

Given the heterogeneity of the included clinical, single-cell, animal, and mechanistic studies, the present narrative review did not attempt to assign formal methodological quality scores. Instead, we developed a three-tier evidence-directness framework to indicate how directly the available evidence supports each specific biological proposition.

Evidence was considered direct when the study population, tissue or model, cellular state, and biological relationship substantially matched the proposition being evaluated. Evidence was considered extrapolated when one or more critical components differed, such as evidence derived from non-HCC tumors, non-hepatic inflammatory diseases, non-diabetic metabolic liver disease, or mechanistic experiments that demonstrated only an individual pathway component without testing the complete proposed relationship.

Level A (direct evidence): evidence that directly addresses the specific proposition in the relevant disease or biological context. This category includes disease-context clinical observations, appropriately annotated human tissue or single-cell studies, and experimental studies directly testing the stated relationship. Level A indicates direct relevance to the proposition but does not necessarily imply causality.

Level B (indirect mechanistic support): evidence supporting one or more mechanistic components of the proposition but obtained from a related disease context, cell state, tissue, or experimental model in which the complete proposed relationship was not directly tested.

Level C (hypothesis-generating evidence): relationships inferred from the integration of multiple indirect observations, biological plausibility, or cross-context extrapolation for which direct disease-specific evidence is currently absent.

Intermediate categories (A–B or B–C) were used only when evidence for a proposition spanned adjacent levels because of substantial differences in study design, biological context, or directness. Importantly, these categories describe the directness and contextual relevance of evidence to a specific proposition rather than the methodological quality of an individual study. Accordingly, a high-quality mechanistic study performed outside the relevant liver/HCC context may provide Level B rather than Level A evidence for a disease-specific proposition.

## 3 Remodeling of hepatic immune homeostasis by metabolic dysfunction

### 3.1 Systemic metabolic stress and hepatocyte injury

When metabolic dysfunction enters the hepatic homeostatic system, the liver is exposed not to a single abnormal metabolic parameter but to a chronic metabolic milieu shaped by obesity, visceral adipose tissue dysfunction, insulin resistance, dyslipidemia, gut microbiota dysbiosis, and altered bile acid metabolism (12, 13). These systemic changes increase hepatic exposure to free fatty acids, glucose, fructose-derived substrates, and gut-derived inflammatory signals (14). Under this condition, hepatocyte lipid accumulation becomes progressively linked to lipotoxicity, mitochondrial dysfunction, oxidative stress, endoplasmic reticulum stress, and the release of damage-associated molecular patterns, thereby providing upstream inflammatory cues during the transition from MASLD to MASH (12–14).T2DM represents a clinically relevant metabolic context in which these processes are further amplified. Insulin resistance, compensatory hyperinsulinemia, hyperglycemia, enhanced adipose tissue lipolysis, and sustained free fatty acid influx may promote hepatic *de novo* lipogenesis, increase mitochondrial burden, and aggravate hepatocyte stress (13, 14, 18, 19). Injured hepatocytes can transmit metabolic pressure to resident and recruited immune cells through cell death signals, altered antigen exposure, and danger-associated signals (12, 20–22).

### 3.2 Myeloid, endothelial, and stromal remodeling

Kupffer cells and monocyte-derived macrophages sense lipid overload, cholesterol-related stimuli, and damage-associated molecular patterns, and subsequently release inflammatory mediators and chemokines, including TNF-α, IL-6, and monocyte-recruiting signals (20, 22, 23). These factors not only sustain local inflammation but also shape the cytokine environment in which adaptive immune cells are activated, retained, or functionally redirected (20–22).

In addition to inflammatory cytokines, metabolic dysfunction alters the spatial and tolerogenic architecture of the liver immune microenvironment. Liver sinusoidal endothelial cells participate in antigen presentation and immune tolerance, whereas hepatic stellate cells, activated fibroblasts, and extracellular matrix deposition may create fibrotic niches and spatial barriers that influence immune cell trafficking and localization (20, 24). Chemokine axes such as CXCL9/CXCL10–CXCR3 and CXCL12–CXCR4 may further regulate the recruitment, positioning, and retention of T cells within inflamed or fibrotic liver tissue (24–26).

### 3.3 Lymphocyte remodeling and the transition toward GZMK-associated CD8^+^ T-cell states

At the same time, cytokines such as IL-6, TGF-β, and IL-10, together with altered glucose, fatty-acid, and amino-acid availability, may influence CD8^+^ T-cell differentiation, effector function, exhaustion-like features, and tissue adaptation (20–22, 26). Metabolic remodeling is not restricted to CD8^+^ T cells but may simultaneously alter other lymphocyte populations, particularly regulatory T cells (Tregs). Tumor-infiltrating Tregs exhibit substantial metabolic flexibility and can maintain suppressive activity in nutrient-restricted, lactate-rich environments, thereby further constraining effector T-cell function and potentially influencing responses to immune checkpoint blockade (27, 28).

Therefore, the impact of T2DM on hepatic immune homeostasis should not be viewed simply as a linear inflammatory response driven by hyperglycemia. Instead, it reflects coordinated remodeling of hepatocyte stress signals, myeloid-cell activation, endothelial and stromal niches, chemokine gradients, cytokine networks, and nutrient availability (20, 25, 26, 29, 30). Collectively, these studies establish that metabolic dysfunction can remodel the hepatic immune and stromal environment, but they do not establish that T2DM preferentially induces or expands a GZMK^+^ CD8^+^ T-cell state. This distinction represents the first major evidence gap in the proposed framework. The unresolved question is therefore not whether metabolic dysfunction alters hepatic immunity, which is well supported, but whether specific diabetic metabolic pressures reproducibly shift hepatic CD8^+^ T-cell differentiation toward GZMK-high transitional states and whether such remodeling precedes or accompanies HCC development. Accordingly, the metabolic and microenvironmental mechanisms summarized above are considered upstream candidate pressures rather than direct evidence for a T2DM–GZMK^+^ CD8^+^ T-cell axis.The established upstream components of metabolic and hepatic immune remodeling, together with the hypothesized transition toward GZMK-associated CD8^+^ T-cell states, are summarized schematically in **Figure 1**.

**Figure 1:**
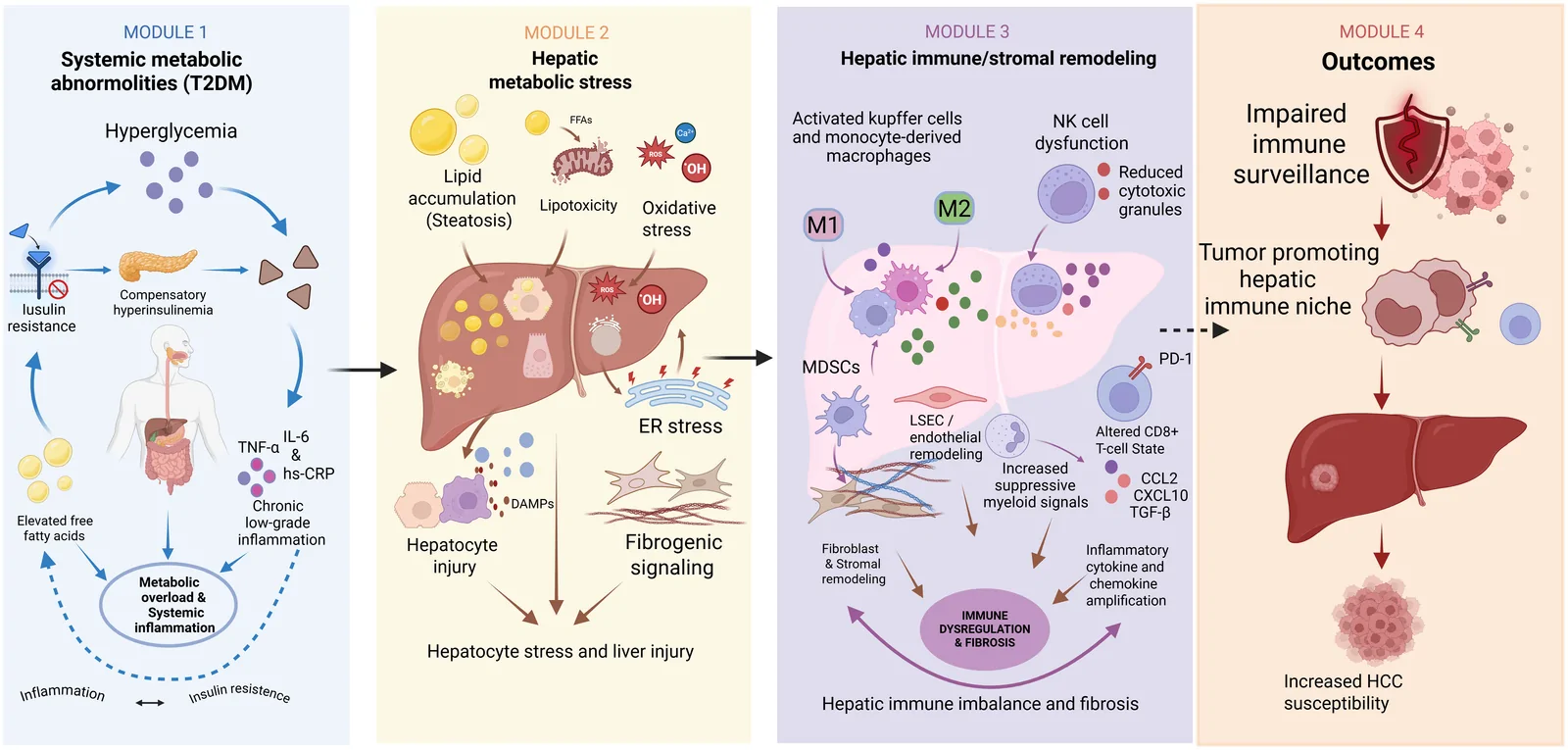
Systemic metabolic dysfunction and remodeling of the hepatic immune microenvironment relevant to HCC susceptibility. Systemic metabolic abnormalities associated with T2DM and related metabolic disorders promote hepatic lipid accumulation, oxidative and endoplasmic reticulum stress, hepatocyte injury, inflammatory signaling, and fibrogenic responses. These processes reshape myeloid, endothelial, stromal, and lymphocyte compartments, generating an altered hepatic immune microenvironment that may impair antitumor surveillance and favor HCC development. The figure emphasizes established or broadly supported metabolic and hepatic immune alterations rather than a GZMK-specific causal pathway.

## 4 Definition and heterogeneity of GZMK^+^ CD8^+^ T cells

Clarifying the phenotypic boundaries of GZMK^+^ CD8^+^ T cells is particularly important in HCC because increased GZMK expression cannot, by itself, distinguish preserved antitumor immunity from transitional exhaustion or inflammation-associated remodeling. Throughout this Review, “GZMK^+^ CD8^+^ T cells” refers broadly to cells with detectable GZMK expression, whereas “GZMK-high CD8^+^ T-cell states” is reserved for populations characterized by relatively elevated GZMK expression in transcriptomic or quantitative profiling analyses. These terms are therefore used according to the analytical context rather than interchangeably. The following section therefore focuses on those phenotypic features that are necessary for interpreting GZMK^+^ CD8^+^ T cells in metabolically altered HCC rather than providing a general overview of GZMK biology.

### 4.1 Molecular features of GZMK and its distinction from classical cytotoxic granzymes

Granzyme K (GZMK) is a serine protease expressed mainly by cytotoxic lymphocytes, including subsets of CD8^+^ T cells and natural killer cells (15, 31). However, GZMK should not be considered functionally equivalent to granzyme B (GZMB) or perforin 1 (PRF1). Classical cytotoxic CD8^+^ T cells typically coexpress GZMB, PRF1, NKG7, and IFNG, supporting direct target cell killing (15, 31, 32). In contrast, GZMK is more commonly associated with nonterminally differentiated states involving inflammatory communication, migration, and tissue adaptation (15, 31). High GZMK expression alone therefore does not indicate enhanced tumor killing. It may instead identify a plastic state in which partial effector capacity coexists with adaptation to persistent stimulation and tissue stress (15, 33). Therefore, GZMK should be understood not simply as a cytotoxic marker, but as a molecular feature of a broader CD8^+^ T-cell state.

### 4.2 Transcriptional phenotype of GZMK^+^ CD8^+^ T cells

Single-cell studies frequently identify GZMK-high CD8^+^ T cells within effector-memory-like or transitional populations (7, 15, 33). These cells commonly express genes related to migration, inflammatory signaling, and partial effector function, including CCL5, CXCR3, and NKG7, but their transcriptional profile differs from that of terminal cytotoxic effectors (15, 33, 34). Across the CD8^+^ T cell differentiation continuum, they are often positioned between activated effector memory states and exhaustion-related lineages (15, 29, 32, 35). They may retain effector features while acquiring adaptations to chronic antigen exposure, inflammatory stress, nutrient availability, and tissue specific signals. GZMK^+^ CD8^+^ T cells are therefore better interpreted as heterogeneous intermediate states rather than as a uniform cytotoxic or terminally exhausted population (7, 15, 17, 29, 32, 35–37).

### 4.3 Relationship with Tem, Tpex, Tex, and Trm states

Within this nomenclature, GZMK^+^ CD8^+^ T cells are used as an operational phenotypic designation rather than to define a discrete or lineage-restricted CD8^+^ T-cell subset. GZMK expression spans multiple differentiation states and may occur in effector-memory, precursor-exhausted, exhausted, and tissue-resident populations depending on the tissue and disease context (15, 17, 32, 33, 35, 37). GZMK-high CD8^+^ T cells frequently occupy intermediate or non-terminally differentiated states, often retaining inflammatory or effector-memory features while exhibiting a less terminally cytotoxic profile than classical GZMB-high effector cells (15, 33, 34). Therefore, GZMK expression alone is insufficient to assign a specific differentiation state, and its interpretation should incorporate progenitor-, exhaustion-, residency-, and cytotoxicity-associated programs (17, 32, 35, 37).

GZMK expression alone is insufficient to define precursor exhausted T cells. A Tpex or stem like exhausted identity requires additional evidence, including maintenance of TCF1 or TCF7, expression of SLAMF6 or CXCR5, self-renewal capacity, and the ability to generate terminally exhausted progeny (5, 32, 35). Although some GZMK-high tumor reactive populations show lower exhaustion and stronger precursor signatures in treatment responsive settings, these findings remain context dependent (33).

GZMK^+^ CD8^+^ T cells should likewise not be classified automatically as terminally exhausted. Terminal exhaustion is associated with sustained inhibitory receptor expression, TOX related programs, restricted effector function, and limited reinvigoration potential (5, 32, 35). GZMK may occur in transitional exhaustion states but is not a defining marker of terminal exhaustion (15, 35). Some GZMK^+^ populations may also acquire tissue-resident memory features, including CD69, ITGAE, or CXCR6, although this relationship varies across tissues and disease contexts (17, 32, 37). In the liver, local retention, antigen exposure, and stromal interactions may further modify these overlapping phenotypes (17, 29, 37). The representative phenotypic and functional features distinguishing these overlapping GZMK-associated CD8^+^ T-cell states are summarized in **Table 1**. This distinction is particularly relevant to HCC, where an apparent increase in GZMK^+^ CD8^+^ T cells could therefore reflect biologically divergent states. Resolving their significance requires paired assessment of GZMK with cytotoxic, progenitor/exhaustion, tissue-retention, and spatial features rather than enumeration of GZMK^+^ cells alone.

**Table 1:** Comparison of GZMK-associated CD8^+^ T-cell states across the differentiation continuum.

CD8^+^ T-cell state	Representative markers	GZMK feature	Distinguishing features
Effector CD8^+^ T cells	GZMB, PRF1, NKG7, IFNG	Variable	Direct cytotoxic killing
Effector-memory-like CD8^+^ T cells	GZMK, CCL5, CXCR3, NKG7	Frequently high	Migration, inflammatory response, partial cytotoxicity
Precursor exhausted/stem-like exhausted CD8^+^ T cells	TCF7, SLAMF6, PDCD1, CXCR5	May be present	Self-renewal and source of terminally exhausted progeny
Transitional exhausted CD8^+^ T cells	GZMK, PDCD1, CXCR3, TOX variable	Often enriched	Intermediate state between effector memory and exhaustion
Terminally exhausted CD8^+^ T cells	TOX, HAVCR2, LAG3, PDCD1, TIGIT	Not necessarily high	Restricted effector function and poor reinvigoration potential
Tissue-resident memory-like CD8^+^ T cells	CD69, ITGAE, CXCR6	Context-dependent	Local immune surveillance and tissue retention

Marker selection is summarized based on studies of CD8^+^ T-cell differentiation, exhaustion, HCC immune microenvironment, and GZMK^+^CD8^+^ T-cell biology (5, 7, 15, 17, 31–35, 37).

### 4.4 Cross-species, model-specific, and clinical-annotation Issues

The nomenclature and phenotypic definition of GZMK^+^ CD8^+^ T cells vary across biological contexts, species, and analytical strategies. Similar populations have been described as GZMK^+^ effector-memory, transitional, precursor-like exhausted, tumor-reactive-like, or non-terminally exhausted CD8^+^ T cells (15, 33, 34). Although these terms are not interchangeable, they may represent overlapping positions along the CD8^+^ T-cell differentiation continuum. Human and mouse GZMK-associated populations may share conserved transcriptional modules but differ in marker combinations, tissue distribution, differentiation trajectories, and functional properties (15, 32, 35). Therefore, conservation of GZMK expression alone should not be interpreted as evidence of equivalent cellular states or functions across species. Cross-species comparisons should instead integrate transcriptional programs, protein phenotype, spatial localization, and functional characteristics, particularly in the metabolically altered HCC context.

Commonly used T2DM mouse models introduce an additional level of uncertainty. The db/db model develops obesity, insulin resistance, and hyperglycemia as a consequence of leptin-receptor deficiency, whereas human T2DM is typically polygenic and clinically heterogeneous (38). The HFD+STZ model combines diet-induced metabolic stress with partial β-cell injury and may reproduce some features of human T2DM, but its phenotype is strongly influenced by diet composition and duration, STZ dose and schedule, age, sex, and genetic background (38). Excessive β-cell injury may also shift the model toward relative insulin deficiency rather than the insulin-resistant state characteristic of earlier human T2DM. Thus, neither model should be assumed to reproduce the human T2DM immune phenotype in its entirety, and GZMK^+^ CD8^+^ T-cell findings should be considered model-dependent until reproduced across complementary metabolic models and validated in human tissues.

Genetic background represents a further potential source of variability. C57BL/6 and BALB/c mice differ in baseline immune architecture and T-cell polarization (39), and they also show strain-dependent susceptibility to diet-induced metabolic dysfunction (40). However, whether hepatic GZMK^+^ CD8^+^ T-cell abundance, differentiation, or metabolic responsiveness differs systematically between these strains has not been established. Strain-dependent immune characteristics should therefore be considered a potential confounder rather than assuming that GZMK-associated findings obtained in one genetic background are universally transferable. Future studies should report strain and substrain, sex, age, diet composition, and metabolic phenotype in detail and, where feasible, reproduce key findings across more than one genetic background.

Translation to human HCC is further limited by incomplete metabolic annotation in existing single-cell datasets. Many representative HCC single-cell studies have primarily characterized tumor immune heterogeneity, immune-state transitions, disease etiology, or treatment response rather than performing prespecified analyses stratified by T2DM status (7, 37, 41). Consequently, diabetes status and related metabolic variables, including glycemic control, duration of diabetes, antidiabetic medication, obesity, and coexisting MASLD/MASH, have not been consistently incorporated into immune-state analyses across available cohorts. The presence of GZMK-high CD8^+^ T-cell states in human HCC therefore cannot currently be attributed specifically to T2DM (7, 37, 41). Future studies should prospectively integrate single-cell and spatial profiling with standardized metabolic annotation, including T2DM status, HbA1c or longitudinal glycemic control, BMI, MASLD/MASH and fibrosis status, and antidiabetic treatment. Ideally, paired tumor, peritumoral, non-tumor liver, and peripheral blood samples should be analyzed to enable transcriptional, protein-level, spatial, and functional validation of GZMK^+^ CD8^+^ T-cell states identified in mouse models.

### 4.5 GZMK expression does not equal extracellular GZMK activity

GZMK mRNA expression, GZMK protein positivity, and extracellular enzymatic activity represent distinct levels of evidence (15, 31). High GZMK mRNA identifies a transcriptional state but does not establish protein abundance, secretion, protease activity, or substrate cleavage (15, 33). Similarly, protein-based assays confirm the presence of GZMK but cannot determine whether it has been released and remains enzymatically active. Evidence of extracellular active GZMK requires demonstration of release and substrate cleavage, ideally together with biological effects that are attenuated by GZMK inhibition (31, 42). Mechanistic claims involving complement activation, PAR-1 or PAR-2 signaling, fibroblast activation, or inflammatory mediator release should therefore be attributed to extracellular active GZMK rather than to GZMK^+^CD8^+^ T-cell abundance alone (15, 31, 42). Most studies in metabolic liver disease and HCC have examined GZMK transcriptional states or protein-positive cells, whereas direct evidence of local extracellular GZMK activity remains limited. Accordingly, this review treats GZMK^+^ CD8^+^ T cells primarily as a candidate immune state and a potential source of GZMK, while extracellular substrate cleavage remains a context-dependent mechanism requiring direct validation.

## 5 Context-dependent functions of GZMK^+^ CD8^+^ T cells

### 5.1 Antitumor immunity and therapeutic responsiveness

The functional significance of GZMK^+^ CD8^+^ T cells is highly context-dependent. In some tumor settings, GZMK-high CD8^+^ T-cell states may participate in antitumor immune responses and therapeutic responsiveness (15, 33, 34, 43). Certain tumor reactive or precursor like populations with relatively high GZMK expression have been linked to improved responses to immune checkpoint blockade (33). In HCC, clinical benefit from anti-PD-1 therapy combined with lenvatinib was associated with enrichment of GZMK^+^CD8^+^ Teff/Tem populations, suggesting that some GZMK-high states may participate in effective antitumor responses (34, 43).

However, GZMK expression does not universally indicate protective immunity. Its functional relevance depends on whether these cells retain proliferative capacity, tumor reactivity, cytotoxic potential, and responsiveness to therapeutic reinvigoration (5, 33, 35, 43). GZMK should therefore be interpreted together with representative cytotoxic, exhaustion-related, progenitor-like, and trafficking markers rather than as an independent indicator of antitumor activity (15, 32–35, 44).

### 5.2 Extracellular active GZMK and complement-dependent inflammatory amplification

GZMK may also contribute to extracellular inflammatory amplification. Unlike GZMB, which is closely associated with classical apoptotic cytotoxicity, GZMK has been described as a tryptase like protease with inflammatory functions (15, 31). Biochemical evidence indicates that active GZMK can cleave complement components C2 and C4 and promote downstream C3 and C5 cascade activation (42).

These findings should not be extrapolated directly from GZMK^+^CD8^+^ T cell abundance. Complement activation requires extracellular release of enzymatically active GZMK and access to relevant substrates. GZMK^+^ CD8^+^ T cells may represent a potential source, but direct evidence of local GZMK mediated complement activation in HCC or metabolic liver disease remains limited. This mechanism should therefore be considered biologically plausible but incompletely validated in these settings (16, 17, 20, 29).

### 5.3 Chronic inflammation, tissue injury, and metabolic liver disease

In metabolically stressed or chronically inflamed liver environments, GZMK^+^ CD8^+^ T cells may acquire a different functional meaning. MASLD, MASH, and T2DM-associated liver injury are characterized by lipid overload, oxidative stress, hepatocyte damage, fibrotic remodeling, altered cytokine networks, and myeloid cell accumulation (12, 14, 20, 22, 24, 30, 45). These factors can reshape the activation threshold, metabolic fitness, survival mode, and differentiation trajectory of CD8^+^ T cells (16, 20, 21, 36, 46, 47).

Under these conditions, GZMK-high CD8^+^ T cells may represent an inflammatory but incompletely cytotoxic state shaped by persistent stimulation, metabolic stress, fibrotic remodeling, and suppressive myeloid signals (16, 17, 20, 36, 41, 46). This possibility is relevant to T2DM-associated HCC because diabetes may influence hepatic CD8^+^ T cell states before and during tumor development rather than acting solely as a systemic comorbidity (3, 4, 16, 36). Expansion of GZMK^+^ CD8^+^ T cells in metabolic liver disease should therefore be interpreted as potential immune remodeling rather than evidence of enhanced cytotoxic antitumor immunity.

### 5.4 Reconciling the context-dependent and apparently contradictory roles of GZMK^+^ CD8^+^ T cells

Apparently contradictory observations have been reported for GZMK^+^ CD8^+^ T cells across tumor and inflammatory contexts. In some studies, GZMK-high effector-memory or non-terminally exhausted CD8^+^ T-cell states are associated with preserved antitumor activity and favorable responses to immune checkpoint blockade (33, 34, 43). In contrast, GZMK-high populations have also been linked to chronic inflammatory remodeling, stromal interaction, or unfavorable clinical features in other disease settings (10, 11, 15). Importantly, these observations do not necessarily indicate intrinsically opposing functions of a single GZMK^+^ CD8^+^ T-cell population. Rather, they may reflect biologically distinct cell states that share GZMK expression but differ in cytotoxic capacity, exhaustion status, tissue localization, and surrounding cellular niches.

HCC provides an illustrative example of this context dependence. Lenvatinib-associated GZMK^+^ CD8^+^ T cells have been reported to display cytotoxic features and to increase in treatment-associated HCC, yet these cells were preferentially localized within the intratumoral stroma rather than uniformly penetrating tumor nests (34). Thus, a GZMK^+^ CD8^+^ T-cell population may retain cytotoxic potential while simultaneously occupying an immune-excluded spatial niche that limits effective tumor-cell engagement. This distinction may help reconcile observations linking GZMK-high states to favorable immune responsiveness with studies implicating similar populations in inflammatory or poorly effective immune microenvironments.

The inconsistent prognostic associations reported for GZMK may likewise arise from methodological and clinical heterogeneity. Bulk RNA-based GZMK expression cannot distinguish whether the signal originates from GZMK^+^ CD8^+^ T cells, other lymphocyte populations, or differences in overall immune infiltration, whereas single-cell, flow-cytometric, or spatial approaches resolve distinct cellular states and tissue compartments. Moreover, GZMK-high CD8^+^ T cells may range from GZMB/PRF1-competent effector-memory populations to PD-1/TOX-associated transitional states, which are unlikely to have equivalent prognostic significance. Spatial localization provides an additional source of variation, because cells within tumor nests, stromal regions, peritumoral liver, or fibrotic niches may differ substantially in access to malignant cells and local regulatory signals (7, 17, 29, 37, 42). Finally, HCC cohorts differ in underlying etiology, fibrosis severity, treatment exposure, metabolic comorbidities, and antidiabetic therapy, and most available studies have not stratified GZMK-associated outcomes according to T2DM status. These sources of heterogeneity may explain why GZMK expression alone has not emerged as a consistent prognostic biomarker.

Their functional meaning should consequently be evaluated by integrating phenotype, spatial localization, neighboring cell populations, and the metabolic inflammatory context rather than by GZMK expression alone (17, 29, 32, 34, 35, 37, 44, 48). Representative disease contexts supporting the biological interpretation of GZMK^+^CD8^+^ T-cell states are summarized in **Table 2**.

**Table 2:** Contextual characteristics and limitations of evidence on GZMK^+^ CD8^+^ T-cell states across disease settings.

Disease/context	Species	Etiology/metabolic context	Cohort/model and tissue	Assay/evidence type	GZMK^+^ CD8^+^ T-cell finding	Directness to HCC/T2DM context	Causal vs associative evidence	Major limitations
Aging/inflammaging	Human	Aging-associated chronic inflammation; non-HCC context	Peripheral blood and aged/inflamed tissues	Cell-state and phenotypic profiling	Clonally expanded, tissue-homing, exhaustion-associated GZMK^+^ CD8^+^ T-cell states	Extrapolated to HCC and T2DM	Primarily observational/associative	Non-HCC setting; heterogeneous tissues; does not establish a role in metabolically altered liver or HCC
Chronic rhinosinusitis with nasal polyps	Human	Chronic non-hepatic inflammatory disease	Nasal-polyp tissue; CXCL12^+^ fibroblast-rich niches	Cell-state profiling and functional interaction studies	GZMK^+^GZMB^-^CXCR4^+^CD8^+^ T cells interact with CXCL12^+^ fibroblasts and are linked to neutrophilic inflammation	Mechanistically informative but extrapolated to HCC/T2DM	Mechanistic evidence within the nasal-polyp context	Non-hepatic disease; hepatic stellate cells/CAFs were not tested; relevance of the CXCR4–CXCL12 interaction to HCC remains unvalidated
HCC	Human	HCC of incompletely or variably annotated etiology; T2DM status generally not prespecified	Tumor and adjacent/peritumoral liver; treatment-associated HCC cohorts	Single-cell, clinical, and spatial/phenotypic analyses	GZMK^+^ effector-memory/transitional CD8^+^ T-cell states are present in HCC and may change with therapy	Direct to HCC; indirect to T2DM-specific HCC	Observational and treatment-associated	Limited metabolic/T2DM annotation; treatment-associated enrichment does not establish causality; GZMK expression alone does not define function
MASH-related HCC	Human	MASH-associated HCC	Liver and tumor tissue from MASH-associated HCC cohorts	Spatial proteomic/immune phenotyping and related tissue-level analyses	GZMK-associated CD8^+^ T-cell states occur together with exhaustion-associated features, including PD-1/LAG-3 expression	Direct to MASH-associated HCC; not T2DM-specific	Observational/associative	T2DM contribution cannot be isolated; cross-sectional immune-state associations do not establish causal remodeling by GZMK^+^ CD8^+^ T cells
Proposed HCC model in the context of T2DM	Not directly established requires human and mouse validation	T2DM, with potentially overlapping but non-equivalent MASLD/MASH	Proposed comparison of diabetic liver, tumor, peritumoral liver, and peripheral blood; longitudinal diabetic HCC models required	Integrated hypothesis based on clinical, single-cell, metabolic, and mechanistic evidence from related contexts	Hypothesized enrichment or redirection of transitional/inflammatory GZMK^+^ CD8^+^ T-cell states under diabetic metabolic stress	Level C/hypothesis-generating; no direct T2DM-stratified evidence	Hypothesis-generating; causal relationship unestablished	No adequately T2DM-stratified longitudinal evidence; confounding by MASLD/MASH, obesity, glycemic control, and antidiabetic therapy; phenotypic and functional validation lacking

## 6 Potential involvement of GZMK^+^ CD8^+^ T cells in hepatic immune microenvironment remodeling

Building on the evidence framework summarized in **Table 3**, this section examines candidate mechanisms through which T2DM-associated metabolic stress may influence hepatic GZMK^+^ CD8^+^ T-cell states, including metabolic pressure, myeloid and stromal niches, and extracellular GZMK activity.

**Table 3:** Evidence gaps and experimental validation priorities for the proposed T2DM–GZMK^+^ CD8^+^ T-cell–HCC framework.

Proposition	Evidence level	Species/disease context	Cohort/model & assay	Evidence nature/directness	Critical evidence gap	Required validation
T2DM/MASLD increases HCC risk	Level A	Human; T2DM and/or MASLD/MASH populations with HCC outcomes	Epidemiological and clinical cohorts; population-level analyses	Direct epidemiological association; associative rather than mechanistically causal	Causal immune mediators remain incompletely defined	Longitudinal metabolic and immune profiling
Metabolic stress reshapes hepatic immune homeostasis	Level A–B	Human and mouse; MASLD/MASH, T2DM-related metabolic stress, and metabolically altered liver	Single-cell, spatial, clinical, and experimental metabolic-liver studies	Direct for general hepatic immune remodeling; indirect for GZMK-specific remodeling	Which metabolic pressures selectively affect GZMK-high states is unknown	Controlled glucose/lipid/amino-acid perturbation; longitudinal liver models
GZMK^+^ CD8^+^ T cells exist in HCC and change after therapy	Level A	Human HCC; metabolic/T2DM status incompletely annotated in most cohorts	HCC tumor/peritumoral tissue; single-cell, spatial, phenotypic, and treatment-associated analyses	Direct to HCC; observational/treatment-associated, not causal; not T2DM-specific	Functional identity varies across niches and treatments	Multidimensional phenotype, TCR clonality and spatial profiling
T2DM induces hepatic GZMK^+^CD8^+^ T-cell expansion	Level C	Proposed in human and mouse T2DM; direct disease-specific evidence lacking	No adequately T2DM-stratified longitudinal human cohort or validated longitudinal model directly testing this proposition	Hypothesis-generating; extrapolated from metabolic-liver and CD8^+^ T-cell studies	No direct T2DM-stratified or longitudinal evidence	T2DM-stratified human liver/HCC cohorts; diabetic mouse models; flow cytometry, scRNA-seq and spatial validation
GZMK^+^ CD8^+^ T cells promote stromal/myeloid remodeling in T2DM-HCC	Level C	Evidence mainly from human non-hepatic inflammatory settings and HCC-associated stromal/myeloid studies	Functional studies in related inflammatory tissues plus spatial/observational HCC evidence	Mechanistic support in related contexts but extrapolated to T2DM-HCC	Current support is mainly extrapolated from non-hepatic or non-GZMK-specific systems	GZMK^+^ CD8^+^ T-cell–macrophage/HSC/CAF co-culture; spatial proximity analysis; CXCR4/CXCL12 or other pathway perturbation
Extracellular GZMK mediates complement/PAR signaling in metabolically altered HCC	Level C	Experimental mechanistic systems; no direct T2DM-HCC demonstration	Biochemical and functional assays of extracellular GZMK activity	Mechanistically supported for GZMK proteolytic activity, but extrapolated to metabolically altered HCC	No direct demonstration of active extracellular GZMK or substrate cleavage in liver lesions	Active-GZMK assays; C2/C4 or PAR cleavage; GZMK inhibition/knockout
GZMK^+^ CD8^+^ T-cell remodeling contributes to increased HCC susceptibility/progression	Level C	Proposed in metabolically altered/T2DM liver and HCC	Indirect observational and mechanistic evidence; no direct causal model	Hypothesis-generating; causality not established	Association has not been shown to be causal	Longitudinal HCC models; GZMK/state-specific perturbation; tumor incidence/burden and immune-remodeling endpoints
GZMK^+^ CD8^+^ T cells predict outcome or therapy response	Level B-C	Human HCC; heterogeneous etiology and metabolic status	Retrospective clinical, bulk/single-cell, treatment-associated and spatial cohorts	Associative candidate biomarker; limited predictive validation	No prospective T2DM-stratified validation	Independent clinical cohorts integrating metabolic status, immune phenotype, spatial location and outcome

Evidence-level definitions: Level A, direct evidence supporting the specific proposition, based on clinical observations and/or experimental mechanistic validation; Level B, indirect but mechanistically supportive evidence derived from related disease settings, cell states, or experimental models without direct validation of the complete proposition; Level C, hypothesis-generating evidence based primarily on biological plausibility, converging observations, or cross-context extrapolation without direct causal validation. Intermediate categories (A–B and B–C) indicate evidence spanning adjacent levels. Evidence levels refer to the specific proposition evaluated and do not imply that all components of the proposed T2DM–GZMK^+^ CD8^+^ T-cell–HCC axis are established in the same disease context.

### 6.1 Metabolic pressure and T-cell remodeling

T2DM-associated metabolic stress may reshape CD8^+^ T-cell differentiation through interconnected nutrient-sensing and transcriptional programs. Altered glucose availability can affect glycolytic fitness and PI3K–AKT–mTORC1 signaling, whereas lipid accumulation and mitochondrial stress may increase oxidative damage and impair metabolic fitness (21). Amino-acid availability provides an additional regulatory layer by influencing nutrient-sensitive and epigenetic programs that shape exhaustion trajectories (46). In parallel, CXCR4–JAK2–STAT3 signaling can promote TOX-associated exhaustion programs (44). Thus, glucose, lipid, amino-acid, and mitochondrial perturbations may converge on the balance among effector, memory-like, and exhausted CD8^+^ T-cell states rather than acting through a single metabolic pathway.

Within this framework, GZMK-high CD8^+^ T cells may represent a non-terminal and incompletely cytotoxic state whose emergence or persistence is shaped by the surrounding metabolic environment (13, 15, 33, 34, 41, 47). In the liver, these metabolic inputs are likely to be further shaped by sinusoidal antigen presentation, hypoxia gradients, bile-acid signaling, hepatocyte-derived danger signals, and fibrotic stromal restriction. Their frequent association with effector-memory and transitional programs further supports the possibility that metabolic perturbations influence the differentiation landscape in which GZMK-high states emerge or persist. However, direct evidence that specific glucose, lipid, amino-acid, or mitochondrial alterations selectively induce hepatic GZMK-high CD8^+^ T cells remains lacking.

### 6.2 Metabolic crosstalk with regulatory T cells

CD8^+^ T-cell remodeling also occurs in the context of metabolic crosstalk with immunosuppressive lymphocytes, particularly Tregs. Tumor-infiltrating Tregs exhibit substantial metabolic flexibility, allowing them to maintain suppressive activity under metabolically restrictive tumor conditions (49). Tumor-derived metabolites may therefore create a metabolic asymmetry in which effector T cells are compromised while Tregs maintain or enhance their suppressive fitness (27, 49). In HCC, ammonia-rich regions favor Treg persistence while impairing CD8^+^ and CD4^+^ effector T cells, and Treg adaptation to ammonia can enhance oxidative metabolism and contribute to resistance to anti-PD-1 therapy (27). Lactate can similarly reinforce Treg stability through MOESIN lactylation and TGF-β/SMAD3 signaling (28). More recently, AARS1-mediated ATF6 lactylation in HCC cells was shown to enhance TDO2-dependent kynurenine production, thereby promoting Treg differentiation and immunotherapy resistance (50). These findings indicate that metabolic stress may impair CD8^+^ T-cell immunity not only through cell-intrinsic metabolic constraints but also by selectively supporting metabolically adapted Tregs (27, 28, 49, 50). Together, these observations support both cell-intrinsic and Treg-mediated routes through which metabolic stress may reshape CD8^+^ T-cell immunity, but their specific contribution to hepatic GZMK-high CD8^+^ T-cell remodeling remains unresolved. Testing these relationships will require controlled metabolic perturbation experiments and longitudinal diabetic liver or HCC models that directly quantify GZMK^+^ CD8^+^ T-cell state transitions while simultaneously assessing changes in metabolically adapted Treg populations.

### 6.3 Myeloid niches

Myeloid cells may regulate the localization and function of GZMK^+^ CD8^+^ T cells, but available evidence does not support a model in which these T cells unidirectionally drive macrophage polarization. A more plausible interpretation is that macrophage-derived chemokines and inhibitory signals shape their recruitment, persistence, and effector maintenance within spatially restricted niches (20, 21).

In HCC, lenvatinib-associated GZMK^+^CD8^+^ Teff/Tem cells were mainly localized in the tumor stroma and recruited through TAM-derived CXCL9, providing relatively direct evidence that macrophage states can influence their positioning (34). TREM2^+^ TAMs have also been shown to restrict CD8^+^ T-cell infiltration and effector maintenance by reducing CXCL9 and enhancing Galectin-1-associated inhibitory signals, although that study did not specifically examine GZMK^+^ cells (48).TAMs should therefore be viewed primarily as potential organizers of GZMK^+^CD8^+^ T-cell niches. Direct reprogramming of macrophages by these T cells remains insufficiently supported. Direct validation will therefore require spatial and functional studies determining whether defined macrophage states selectively recruit, retain, or reprogram GZMK^+^ CD8^+^ T cells in metabolically altered HCC.

### 6.4 Fibroblast and hepatic stellate cell niches

Stromal retention may provide another link between GZMK^+^ CD8^+^ T cells and hepatic immune remodeling. In nasal polyps, GZMK^+^ CD8^+^ T cells interact with CXCL12^+^ fibroblasts through the CXCR4–CXCL12 axis and promote neutrophil chemokine release (11). This finding shows that GZMK^+^ CD8^+^ T cells can participate in fibroblast-associated inflammatory niches, but its relevance to liver fibrosis and HCC remains extrapolative.

Hepatic stellate cells and HCC-associated cancer-associated fibroblasts form extracellular matrix barriers and chemokine-rich niches that can influence CD8^+^ T-cell infiltration and retention (17, 24, 29). Whether they directly retain GZMK^+^ CD8^+^ T cells through CXCR4–CXCL12 signaling has not been established. Current evidence therefore supports a possible stromal-retention model rather than the conclusion that GZMK^+^ CD8^+^ T cells directly drive hepatic fibrosis. This hypothesis could be tested using spatial colocalization, ligand–receptor analysis, and GZMK^+^ CD8^+^ T-cell–HSC/CAF co-culture with CXCR4–CXCL12 perturbation.

### 6.5 Extracellular GZMK: established activities and potential relevance to HCC

Beyond its intracellular expression, enzymatically active extracellular GZMK has been shown to exert pro-inflammatory proteolytic functions in experimental systems outside HCC and metabolic liver disease. In particular, GZMK can cleave complement components C2 and C4 and promote downstream complement activation, and extracellular GZMK has also been implicated in PAR-dependent inflammatory signaling (15, 31, 42). These findings establish extracellular GZMK as a potential inflammatory protease but do not demonstrate that the same mechanisms operate in metabolically stressed liver tissue or HCC.

Complement activation itself is relevant to hepatic inflammation, immune-cell recruitment, fibrosis, and tumor-associated inflammatory remodeling (20, 22, 23, 51). However, direct evidence showing extracellular GZMK release, local complement cleavage, or GZMK-dependent PAR signaling in T2DM-, MASLD/MASH-, or HCC-associated liver tissue is currently lacking. Thus, a model in which GZMK^+^ CD8^+^ T cells release active GZMK and amplify complement- or PAR-dependent inflammatory remodeling in hepatic stromal or myeloid niches should be regarded as a hypothesis-generating, Level C mechanism that requires direct experimental validation. Validation will require direct measurement of extracellular active GZMK and relevant cleavage products in liver lesions, together with inhibition or loss-of-function experiments demonstrating GZMK-dependent effects.

These candidate relationships among metabolic pressure, immune and stromal niches, and extracellular GZMK activity are summarized in **Figure 2**.

**Figure 2:**
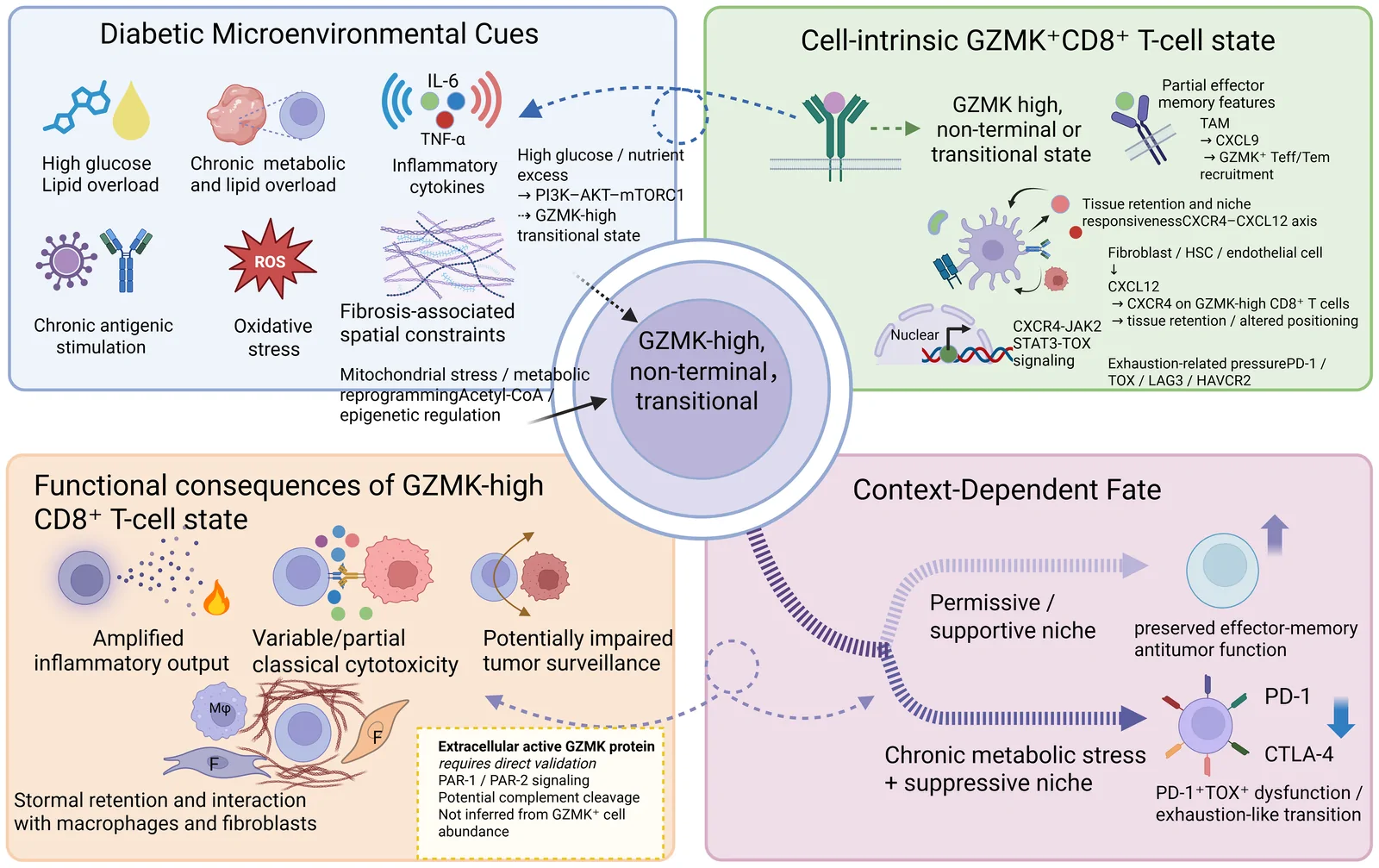
Context-dependent plasticity of GZMK-high CD8^+^ T-cell states in the metabolically altered liver microenvironment. Metabolic and inflammatory cues may contribute to the emergence or persistence of a non-terminal, transitional GZMK-high CD8^+^ T-cell state with partial effector-memory, tissue-retention-related, and exhaustion-associated features. Depending on the surrounding immune and stromal context, these cells may retain antitumor effector functions or shift toward inflammatory, poorly cytotoxic, and dysfunctional states. Extracellular GZMK activity should be distinguished from the abundance of GZMK-expressing cells and requires direct functional validation. Solid arrows indicate experimentally supported or relatively well-established relationships, whereas dashed arrows indicate proposed or hypothesis-generating mechanisms that remain to be directly validated, particularly in T2DM-associated or metabolically driven HCC.

## 7 Evidence gaps and experimental priorities for the proposed T2DM–GZMK^+^ CD8^+^ T-Cell–HCC framework

Using the evidence-directness framework defined in Section 2.2, we next identify the major unresolved transitions in the proposed metabolic dysfunction–GZMK^+^ CD8^+^ T-cell–HCC framework and the experimental approaches required to test them (**Table 3**).

### 7.1 Does T2DM increase hepatic GZMK^+^ CD8^+^ T cells across liver disease stages?

The priority is to determine whether T2DM is associated with a reproducible increase in hepatic GZMK^+^ CD8^+^ T cells before and during HCC development. Future studies should compare GZMK^+^ CD8^+^ T cells across a staged liver disease spectrum, including healthy or non-diseased liver, isolated T2DM, MASLD, MASH, MASH-associated HCC, and non-metabolic HCC (2–4, 13, 14, 19, 20). Such a design would help clarify whether the enrichment of GZMK^+^ CD8^+^ T cells is specifically related to metabolic liver injury, tumor development, or a broader inflammatory HCC phenotype.

In human cohorts, analyses should carefully control for potential confounders, including age, sex, BMI, antiviral status, cirrhosis severity, antidiabetic medication, tumor stage, and previous treatment exposure (2–4, 14, 19, 20, 45). GZMK^+^ CD8^+^ T cells should be quantified not only as a proportion of total CD8^+^ T cells, but also as tissue density and spatial distribution in tumor, peritumoral, and non-tumor liver regions. Flow cytometry, single-cell profiling, and spatial tissue analysis could be combined to distinguish GZMK mRNA expression, GZMK protein positivity, and tissue localization (13, 19, 32, 35, 45, 52). Longitudinal metabolic liver disease models could determine whether these cells emerge before tumor initiation or during HCC progression (13, 19, 45).

### 7.2 Are Hepatic GZMK^+^ CD8^+^ T cells protective effectors or pathology-associated remodeling cells?

The second priority is to determine whether hepatic GZMK^+^ CD8^+^ T cells represent preserved antitumor immunity, dysfunctional inflammatory remodeling, or a transitional state between these two possibilities. Because GZMK expression alone is insufficient to define CD8^+^ T cell function, future studies should adopt multidimensional phenotyping strategies rather than relying on single-marker analysis (12, 22, 23, 30, 45).

A protective effector-like state would be supported by preserved expression of cytotoxic molecules, including GZMB, PRF1, and GNLY, together with effector cytokines or chemokines such as IFNG, TNF, and CCL5 (22, 26, 33, 35). By contrast, a metabolically constrained or pathology-associated state may be characterized by high GZMK expression with reduced GZMB or PRF1 together with increased PDCD1, TOX or tissue-retention markers (11, 13, 23–26, 29, 30, 45). Stem-like or progenitor-exhausted features should also be evaluated using markers such as TCF7 and SLAMF6 (25, 26, 35). TCR clonotype tracking may help distinguish antigen-driven expansion from inflammation-associated recruitment or tissue retention (32, 35, 52).

### 7.3 Does extracellular GZMK have functional activity in liver lesions?

The third priority is to determine whether GZMK is merely a marker of a specific CD8^+^ T cell state or an active extracellular mediator that contributes to hepatic inflammation, stromal remodeling, complement activation, or tumor progression. This distinction is essential because GZMK mRNA expression and GZMK protein positivity do not necessarily demonstrate extracellular enzymatic activity (15, 31, 42).

Future studies should directly detect active extracellular GZMK in liver lesions and assess its downstream substrates or cleavage products. Relevant readouts include complement cleavage and inflammatory or stromal responses (31, 42). Functional perturbation experiments are also required. These may include GZMK inhibition, recombinant-protein experiments, and genetic loss-of-function models (31, 42).

*In vitro* co-culture systems could further test whether extracellular GZMK directly acts on hepatic stellate cells, cancer-associated fibroblasts, macrophages, LSECs, tumor cells, or dendritic cells (11, 24). *In vivo*, the key endpoint should be whether GZMK blockade or deletion alters tumor growth, inflammatory remodeling, complement activation, fibrosis-associated stromal responses, or antitumor immune surveillance. Only these functional experiments can determine whether GZMK acts as a mechanistic effector rather than a correlative marker in T2DM-associated HCC.

### 7.4 Does spatial positioning determine the function of GZMK^+^ CD8^+^ T cells?

The fourth priority is to determine whether the function of GZMK^+^ CD8^+^ T cells depend on their spatial niche within the liver and tumor microenvironment. GZMK^+^ CD8^+^ T cells located in tumor nests and close to dendritic cells may represent active antitumor immune responses, whereas cells retained in stromal or fibrotic regions may be associated with chronic inflammation, immune exclusion, or suppressive remodeling (17, 33, 34, 37). Spatial analyses should examine the relationships of GZMK^+^ CD8^+^ T cells with tumor, myeloid, stromal, and endothelial compartments (11, 13, 22, 32, 33, 35, 45, 52).

Therefore, future studies should avoid treating GZMK^+^CD8^+^ T cell abundance as a standalone indicator. Instead, GZMK^+^ CD8^+^ T cells should be interpreted together with their cytotoxic state, exhaustion state, TCR clonality, extracellular GZMK activity, and spatial relationships with tumor, myeloid, stromal, and endothelial compartments (11–13, 22–26, 29, 30, 32, 33, 35, 45, 52).

This evidence-stratified framework is intended to distinguish association from causation and to clarify whether GZMK^+^ CD8^+^ T cells function as antitumor effectors, markers of pathological remodeling, or sources of biologically active extracellular GZMK in metabolically altered HCC.

### 7.5 Does GZMK^+^ CD8^+^ T-cell remodeling causally influence HCC susceptibility or progression?

The final and most stringent question is whether GZMK^+^ CD8^+^ T-cell remodeling contributes causally to HCC initiation or progression rather than merely accompanying metabolic liver injury or tumor development (3, 16, 34, 41). Longitudinal diabetic HCC models would be needed to establish the temporal sequence between metabolic dysfunction, the emergence of GZMK-high CD8^+^ T-cell states, and tumor formation (13, 20, 41). Functional studies should then determine whether selective depletion, genetic perturbation, adoptive transfer, or manipulation of relevant GZMK-associated states alters HCC incidence, tumor burden, immune surveillance, or stromal remodeling. Comparison with non-metabolic HCC models would further help determine whether any observed effect is specific to the metabolically altered liver environment. Until such experiments are performed, increased HCC susceptibility should not be attributed causally to GZMK^+^ CD8^+^ T-cell remodeling.

## 8 Clinical translation: biomarkers and therapeutic strategies

The translational value of GZMK^+^ CD8^+^ T cells in T2DM-associated HCC should be interpreted cautiously. Current evidence does not support GZMK expression alone as a clinically actionable marker. A more appropriate strategy would be to develop a composite immune-state biomarker integrating GZMK^+^CD8^+^ T-cell abundance, cytotoxic and exhaustion-related phenotypes, spatial localization, glycemic status, and antidiabetic treatment (15, 29, 32, 36, 53). The relationship between T2DM and immune checkpoint inhibitor (ICI) efficacy appears heterogeneous and should not be interpreted as uniformly detrimental or beneficial across malignancies. In melanoma, T2DM has been associated with differences in ICI outcomes, although these observations cannot be directly extrapolated to HCC (54). Such variability may reflect differences in tumor type, treatment regimen, glycemic control, obesity, diabetes severity, and exposure to glucose-lowering medications. Importantly, T2DM itself represents a heterogeneous metabolic condition: patients with well-controlled diabetes may differ substantially from those with persistent hyperglycemia, marked insulin resistance, or advanced metabolic liver disease. These variables are rarely captured simultaneously in retrospective immunotherapy cohorts, making it difficult to isolate the independent contribution of diabetes to treatment response. HCC-specific evidence remains limited and likewise does not support a simple relationship between metabolic therapy and ICI efficacy. For example, metformin use was not associated with improved survival outcomes in patients with T2DM receiving atezolizumab plus bevacizumab (53). Therefore, diabetes status, glycemic control, antidiabetic treatment, and underlying metabolic liver disease should be considered as distinct but interacting variables when evaluating ICI responsiveness in HCC.

In HCC, enrichment of GZMK^+^CD8^+^ Teff/Tem cells following anti-PD-1 plus lenvatinib treatment has been associated with favorable therapeutic responses, while higher GZMK expression has been linked to improved outcomes in some cohorts (34, 43). Spatiotemporal single-cell studies have also identified GZMK-high populations with tumor-reactive-like, circulating, or less terminally exhausted features during PD-1 blockade (35). These findings support the potential use of GZMK^+^ CD8^+^ T cells as dynamic response-associated markers, but they do not demonstrate that these therapies act directly through GZMK. Their stromal localization and association with CXCL9-positive tumor-associated macrophages further suggest that their abundance may reflect a specific immune niche rather than enhanced tumor killing alone (23). In T2DM-associated HCC, no direct evidence currently shows that circulating or intratumoral GZMK^+^CD8^+^ T-cell abundance independently predicts survival, recurrence, or immunotherapy benefit. Prospective validation should therefore evaluate these cells together with cytotoxicity, exhaustion, spatial, and metabolic variables rather than as a single prognostic marker (17, 35, 41, 45, 55). To operationalize such a composite biomarker, a core tissue panel could include CD8 and GZMK to identify the population of interest, GZMB and/or PRF1 to assess cytotoxic competence, PD-1 and TOX to capture exhaustion-associated features, TCF1/TCF7 to identify progenitor-like states, and CXCR4 to assess trafficking or stromal-retention potential (15, 32, 35, 44). Discovery studies could integrate single-cell RNA sequencing, TCR sequencing, and spatial transcriptomics, whereas subsequent validation should prioritize clinically scalable platforms, including multiplex immunofluorescence or immunohistochemistry in tissue and multiparameter flow cytometry in peripheral blood (7, 25, 29, 37, 41). Prognostic validation could initially be performed in resected HCC cohorts with paired tumor and peritumoral liver tissues and recurrence or survival follow-up, whereas predictive validation should involve prospective advanced-HCC cohorts receiving immune-based systemic therapy, with stratification by T2DM status, glycemic control, underlying liver disease, and antidiabetic treatment. Candidate signatures should ultimately be tested in independent validation cohorts.

Extracellular active GZMK represents a second potential translational direction. Experimental studies indicate that it can promote PAR-dependent inflammatory mediator release and complement cleavage, providing a rationale for selective extracellular inhibition (42, 56). Potential approaches include small-molecule or peptide protease inhibitors and neutralizing antibodies, although GZMK-directed pharmacological development remains at an early preclinical stage. A recent study in lung adenocarcinoma used pharmacological GZMK inhibition and showed enhanced antitumor activity when GZMK blockade was combined with anti-PD-1 therapy in immunocompetent and humanized mouse models; however, this evidence is currently restricted to a non-HCC setting and does not establish therapeutic efficacy in metabolically associated liver cancer (57). Specificity represents an important translational challenge because inhibition of related serine proteases could produce off-target biological effects, while systemic GZMK inhibition might also interfere with physiological immune functions. Extracellularly restricted inhibitors or neutralizing approaches may theoretically minimize interference with intracellular GZMK-associated immune functions, but their selectivity, tissue penetration, pharmacokinetics, and safety remain to be established. In T2DM-associated liver lesions or HCC, active GZMK release, local substrate cleavage, and inhibition-sensitive inflammatory effects have not yet been demonstrated. Therapeutic blockade should therefore remain a preclinical concept until target engagement, disease relevance, specificity, and safety are established.

A third direction is modulation of the immune-metabolic niches that shape GZMK^+^ CD8^+^ T-cell localization and function. The CXCR4–CXCL12 axis may contribute to T-cell positioning or retention within fibrotic and stromal niches (24–26, 44), whereas CXCL9-expressing TAMs have been associated more directly with the recruitment and stromal localization of GZMK^+^ CD8^+^ T cells in HCC (34). Extracellular GZMK-mediated complement activation may provide an additional link to inflammatory amplification (15, 31). These observations suggest that translation should focus less on simply increasing or eliminating GZMK^+^ CD8^+^ T cells and more on determining which niches support antitumor activity, inflammatory persistence, or ineffective immune activation. Importantly, niche-directed interventions should be evaluated in combination with established HCC treatment backbones rather than as isolated strategies. In preclinical HCC models, combined CXCR4 and PD-1 blockade inhibited tumor growth, prolonged survival, increased intratumoral CD8^+^ T-cell accumulation and activation, and reprogrammed dendritic-cell responses, providing proof of principle that targeting the CXCL12–CXCR4 axis can enhance checkpoint blockade (58). These findings do not demonstrate a GZMK-dependent mechanism, but they provide a tractable framework for testing whether CXCR4 blockade can alter the spatial localization or functional state of GZMK-high CD8^+^ T cells in HCC.

For clinical translation, such strategies should ultimately be considered in the context of established immune-based HCC regimens. Atezolizumab plus bevacizumab has demonstrated improved clinical outcomes over sorafenib in unresectable HCC, whereas sintilimab plus the bevacizumab biosimilar IBI305 has shown superior progression-free and overall survival compared with sorafenib in the ORIENT-32 trial (59). Rather than immediately adding a niche-directed agent to these regimens clinically, an appropriate translational sequence would be to first determine whether GZMK-high/CXCR4-associated or stromally restricted immune states correlate with treatment response, then test mechanistically supported interventions in metabolically stratified HCC models, and only thereafter consider biomarker-enriched early-phase combination studies. Serial tissue and blood sampling could determine whether treatment alters the spatial distribution and functional phenotype of GZMK^+^ CD8^+^ T cells rather than merely changing their overall abundance.

Metformin and SGLT2 inhibitors should be considered metabolic modifiers rather than GZMK-targeted therapies. By improving systemic and hepatic metabolic conditions and modulating inflammation, these agents may indirectly influence CD8^+^ T-cell fitness and differentiation. However, direct evidence that their effects in HCC are mediated through changes in GZMK^+^ CD8^+^ T-cell abundance or function is currently lacking (53, 55, 60–62).

Clinical translation should therefore prioritize stepwise composite biomarker development and independent validation, direct demonstration of extracellular GZMK target engagement before therapeutic inhibition, and mechanistically guided evaluation of immune-metabolic niche interventions alongside established HCC treatment backbones. Prospective studies should integrate metabolic indicators, peripheral and intratumoral immune phenotypes, spatial profiles, treatment response, recurrence, and survival. This approach preserves the potential translational relevance of GZMK^+^ CD8^+^ T cells without interpreting their abundance as evidence of GZMK-mediated pathology. The proposed translational framework and its potential clinical applications are summarized in **Figure 3**.

**Figure 3:**
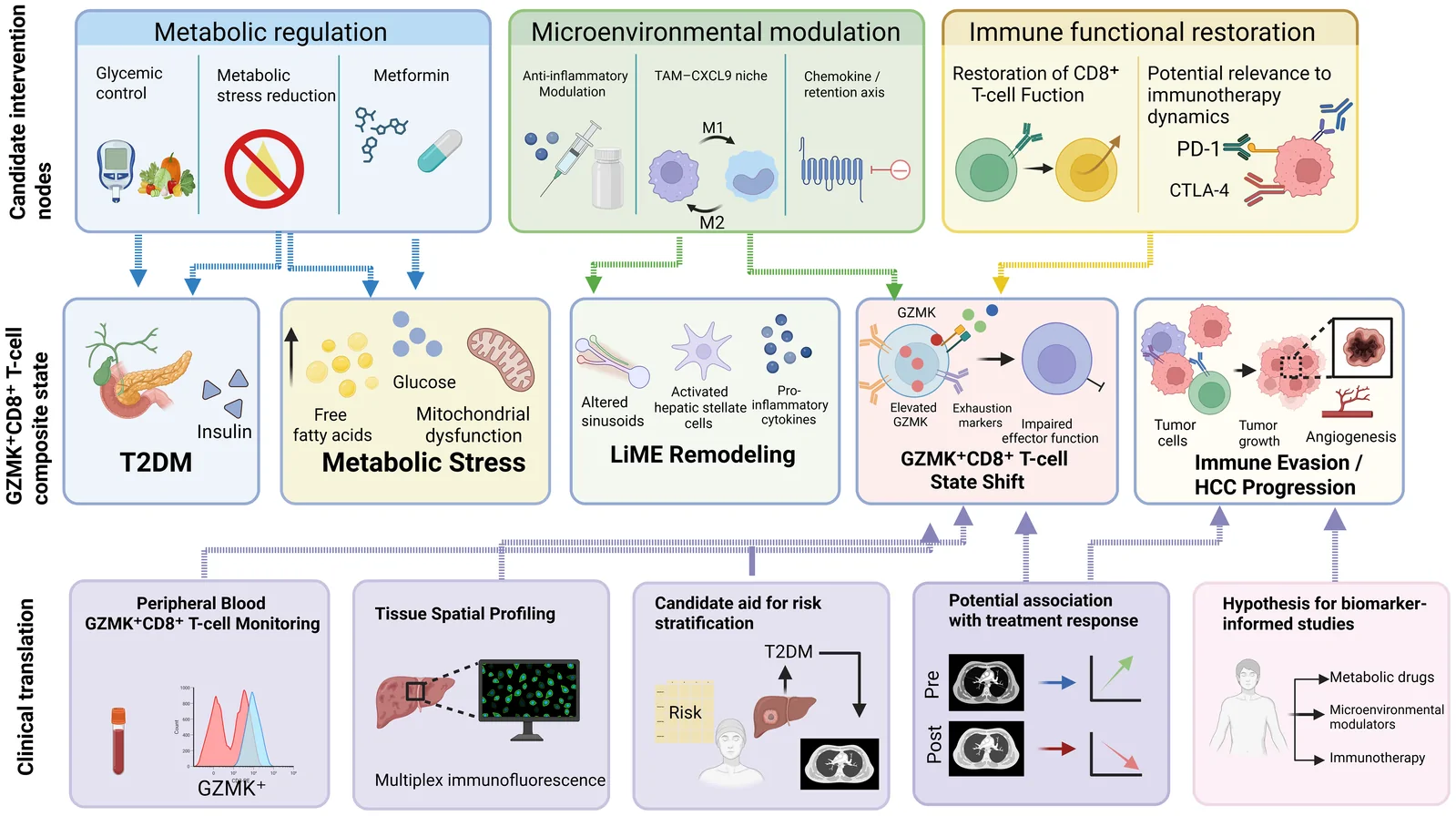
Translational framework for evaluating GZMK^+^ CD8^+^ T-cell states in T2DM-associated HCC. Candidate strategies include metabolic regulation, microenvironmental modulation, and restoration of CD8^+^ T-cell function. Potential applications include peripheral blood monitoring, tissue spatial profiling, risk stratification, treatment-response assessment, and biomarker-informed studies, all of which require prospective validation.

## 9 Discussion

Metabolic dysfunction may influence HCC through overlapping but non-equivalent clinical contexts, including T2DM and MASLD/MASH. T2DM should therefore be considered as a metabolic comorbidity and modifier of the hepatic immune environment rather than as synonymous with MASLD/MASH-associated HCC. Epidemiological evidence supports associations among T2DM, metabolic liver disease progression, and increased HCC risk, whereas the immune mechanisms connecting these conditions remain incompletely defined (2–4). Within this interface, GZMK^+^ CD8^+^ T cells provide a potentially informative lens through which to examine metabolically associated CD8^+^ T-cell remodeling.

Current evidence supports GZMK^+^ CD8^+^ T cells as heterogeneous and context-dependent immune states rather than a stable lineage or uniformly cytotoxic population. Their biological significance is determined by accompanying cytotoxic, progenitor/exhaustion, trafficking, and spatial programs (11, 32–35). Extracellular GZMK activity, however, represents a mechanistically distinct level of evidence that requires direct functional validation. Accordingly, neither GZMK expression nor cell abundance alone establishes a specific functional consequence (15, 31, 42).

The proposed relationship between T2DM-associated metabolic stress and hepatic GZMK^+^ CD8^+^ T-cell remodeling therefore remains a testable hypothesis rather than an established causal pathway. Future studies should integrate T2DM-stratified clinical cohorts with multidimensional phenotyping, spatial profiling, TCR tracking, metabolic characterization, and direct assays of extracellular GZMK activity to determine whether these cells represent protective antitumor states, markers of pathological immune remodeling, or functionally relevant mediators in metabolically associated HCC.
